# Risk Factors for Impaired Cellular or Humoral Immunity after Three Doses of SARS-CoV-2 Vaccine in Healthy and Immunocompromised Individuals

**DOI:** 10.3390/vaccines12070752

**Published:** 2024-07-08

**Authors:** Jae-Hoon Ko, Choon-Mee Kim, Mi-Seon Bang, Da-Yeon Lee, Da-Young Kim, Jun-Won Seo, Na-Ra Yun, Jin-Young Yang, Kyong-Ran Peck, Kyo-Won Lee, Sung-Hoon Jung, Hyun-Jin Bang, Woo-Kyun Bae, Tae-Jong Kim, Kyeong-Hwan Byeon, Sung-Han Kim, Dong-Min Kim

**Affiliations:** 1 Division of Infectious Diseases, Department of Medicine, Samsung Medical Center, Sungkyunkwan University School of Medicine, Seoul 06351, Republic of Korea; jaehoon.ko@gmail.com (J.-H.K.); jinyoung89.yang@samsung.com (J.-Y.Y.); kr.peck@samsung.com (K.-R.P.); 2 Department of Premedical Science, College of Medicine, Chosun University, Gwangju 61452, Republic of Korea; choonmeekim@chosun.ac.kr; 3 Department of Internal Medicine, College of Medicine, Chosun University, Gwangju 61452, Republic of Korea; ktandms@gmail.com (M.-S.B.); barody222@gmail.com (D.-Y.L.); dayz02@hanmail.net (D.-Y.K.); kaist-105@hanmail.net (J.-W.S.); shine@chosun.ac.kr (N.-R.Y.); 4 Division of Transplantation, Department of Surgery, Samsung Medical Center, Sungkyunkwan University School of Medicine, Seoul 06351, Republic of Korea; kw1980.lee@samsung.com; 5 Department of Internal Medicine, Chonnam National University Medical School and Hwasun Hospital, Hwasun 58128, Republic of Korea; shglory@hanmail.net (S.-H.J.); daunjin@gmail.com (H.-J.B.); drwookyun@gmail.com (W.-K.B.); 6 Department of Rheumatology, Chonnam National University Medical School, Gwangju 61469, Republic of Korea; ktj1562@jnu.ac.kr; 7 Department of Parasitology and Tropical Medicine, Chonnam National University Medical School, Gwangju 61469, Republic of Korea; eidanus@naver.com; 8 Department of Infectious Diseases, Asan Medical Center, University of Ulsan College of Medicine, Seoul 05505, Republic of Korea; kimsunghanmd@hotmail.com

**Keywords:** COVID-19 vaccine, immunosuppressed host, interferon-gamma release tests, neutralizing antibody, omicron variant

## Abstract

**Background:** We aimed to identify the risk factors for impaired cellular and humoral immunity after three doses of the SARS-CoV-2 vaccine. **Methods:** Six months after the third vaccine dose, T-cell immunity was evaluated using interferon-gamma release assays (IGRAs) in 60 healthy and 139 immunocompromised (IC) individuals, including patients with hematologic malignancy (HM), solid malignancy (SM), rheumatic disease (RD), and kidney transplantation (KT). Neutralizing antibody titers were measured using the plaque reduction neutralization test (PRNT) and surrogate virus neutralization test (sVNT). **Results:** T-cell immunity results showed that the percentages of IGRA-positive results using wild-type/alpha spike protein (SP) and beta/gamma SP were 85% (51/60) and 75% (45/60), respectively, in healthy individuals and 45.6% (62/136) and 40.4% (55/136), respectively, in IC individuals. IC with SM or KT showed a high percentage of IGRA-negative results. The underlying disease poses a risk for impaired cellular immune response to wild-type SP. The risk was low when all doses were administered as mRNA vaccines. The risk factors for an impaired cellular immune response to beta/gamma SP were underlying disease and monocyte%. In the sVNT using wild-type SP, 12 of 191 (6.3%) individuals tested negative. In the PRNT of 46 random samples, 6 (13%) individuals tested negative for the wild-type virus, and 19 (41.3%) tested negative with omicrons. KT poses a risk for an impaired humoral immune response. **Conclusions:** Underlying disease poses a risk for impaired cellular immune response after the third dose of the SARS-CoV-2 vaccine; KT poses a risk for impaired humoral immune response, emphasizing the requirement of precautions in patients.

## 1. Introduction

Coronavirus disease (COVID-19) is a communicable infectious disease of the respiratory system caused by the Severe Acute Respiratory Syndrome coronavirus 2 (SARS-CoV-2) that caused a global pandemic [1]. Vaccines to prevent SARS-CoV-2 infection are considered the most effective means of suppressing its spread and to prevent severe forms of disease. After SARS-CoV-2 infection, most patients produce neutralizing antibodies and antibodies against the receptor binding domain (RBD) of the viral spike protein (SP) [2]. Among non-survivors of severe COVID-19 disease, there was attenuated IgG response, compromised Fcγ receptor binding, and Fc effector activity [3].

Patients who have recovered from COVID-19 and individuals who have received a COVID-19 vaccination develop CD4^+^ and CD8^+^ T-cell immune responses specific to SARS-CoV-2, suggesting the possibility of durable T-cell immune responses [4,5].

In a study involving 3593 individuals who had received COVID-19 vaccinations from Pfizer BionNTech (BNT162b2), Moderna (mRNA-1273), Sinovac (CoronaVac), and Sinopharm (BBIBP-CorV), those with and without an antibody response to SARS-CoV-2 SP were compared [6]. Seroconversion against the SARS-CoV-2 RBD of the SP was observed in 84% of individuals, while a poor immune response was associated with male sex, age ≥65 years, and recent chemotherapy [6].

The immunogenicity and efficacy of COVID-19 vaccines are lower in immunocompromised (IC) individuals compared to the general population. The proportion of immune non-responders were higher among those with solid organ transplant recipients (18–100%), hematological malignancy (14–61%), those with cancers (2–36%), and those on hemodialysis (2–30%) [7]. In patients with chronic kidney disease or those with kidney transplants, the mean antibody concentration levels were lower than healthy individuals after vaccination with two doses of the mRNA-1273 COVID-19 vaccine [8]. Based on the data of patients with cancers having received three or four doses of the mRNA vaccine, those with three doses of the vaccine had lower antibody levels than those with four doses and an overall lower cell-mediated response [9].

However, few studies have investigated the risk factors for impaired humoral or cellular immune responses [3,6,10,11]. Therefore, this study aimed to identify the risk factors for impaired humoral and cellular immune responses in healthy individuals and patients with IC.

## 2. Materials and Methods

### 2.1. Study Subjects

This multicenter observational study was conducted with the approval of the institutional review board of each center. The study subjects included 123 healthy subjects and 233 subjects with solid malignancy (SM), hematologic malignancy (HM), or rheumatic disease (RD) and patients who underwent kidney transplantation (KT) who had received three homologous or heterologous doses with mRNA (Pfizer, Pfizer and BioNTech, Puurs Belgium, Cambridge, MA, USA), adenovirus (AstraZeneca, AstraZeneca, Cambridge, UK), or other (Janssen, Janssen Biotech, Raritan, NJ, USA) vaccines.

### 2.2. Sample Collection

Blood samples were collected from both healthy individuals and IC patients 3–6 months after the third dose of the SARS-CoV-2 vaccine.

### 2.3. Interferon-Gamma Releasing Assay

To investigate the cellular immune responses in both healthy and IC patients after the third dose of the anti-SARS-CoV-2 vaccine, an interferon-gamma (IFN-γ) releasing assay (IGRA) was performed using an enzyme-linked immunosorbent assay (ELISA; Covi-FERON ELISA, SD Biosensor, Suwon-si, Gyeonggi-do, Republic of Korea). The IFN-γ levels induced by SARS-CoV-2 wild-type/alpha SP and SARS-CoV-2 beta/gamma SP have been measured [10]. Based on the 0.25 U/mL cutoff recommended by the manufacturer, participants were categorized into positive and negative groups, and the risk factors were analyzed by comparing participants’ characteristics.

### 2.4. Neutralizing Antibody Assay

To assess the neutralizing antibody titer against SARS-CoV-2 in both healthy and IC after three doses of the vaccine, a plaque reduction neutralization test (PRNT) was performed using Vero E6 cells (*Cercopithecus aethiops* kidney epithelial cells, CRL-1586, ATCC). The neutralizing antibody titer was measured against the SARS-CoV-2 wild-type (V clade [B lineage], isolated hCoV-19/South Korea/KUMC01/2020, GISAID accession no. EPI_ISL_413017) and omicrons (GR clade [B.1.1.529 lineage]), and isolated hCoV-19/Korea/KDCA447321/2021, Accession no. NCCP43408) was purchased from the Korea Disease Control and Prevention Agency. PRNT_50_, the level at which SARS-CoV-2 viral growth is suppressed by 50%, was defined as the neutralizing antibody titer [12]. Participants were categorized into positive and negative groups using a cutoff of 1:8, and risk factors were analyzed by comparing participants’ characteristics [13].

### 2.5. Neutralizing Antibody Titer Analysis Using the GenBody Rapid Kit

For the surrogate virus neutralizing test (sVNT), the GenBody fluorescence immunoassay (FIA) COVID-19 NAb kit (GenBody, Cheonan-si, Chungcheongnam-do, Republic of Korea) was used. The GenBody sVNT is an FIA used to measure the inhibition of RBD-ACE2 binding based on the antibody-mediated blockage of the interaction between ACE2 and wild-type SARS-CoV-2 SP. Following the manufacturer’s guideline, a cutoff ≥30% (GenBody) was applied.

### 2.6. Statistical Analysis

Six months after the third dose of the SARS-CoV-2 vaccine, healthy and IC individuals were categorized into positive and negative groups based on their PRNT_50_, sVNT, and IFN-γ responses against the viral SP (original and variant strains). The χ^2^ test was used to analyze the results, accounting for factors such as sex, vaccine type, underlying disease, median age, body mass index (BMI), and laboratory test results.

To analyze the risk factors, odds ratios (ORs; 95% confidence interval [CI]) and *p*-values were calculated based on binary logistic regression analysis for PRNT_50_, sVNT, and IFN-γ responses against viral SP (original and variants). Subsequently, a multivariate logistic regression analysis was applied to items with a significant *p*-value in the binary logistic regression analysis to estimate the OR (95% CI) and *p*-value. The level of significance was set at *p* < 0.05. Prism version 8.0.1 (Graphpad Software, San Diego, CA, USA) and SPSS version 26 (IBM, Amonk, NY, USA) were used to analyze the collected data.

## 3. Results

The initial number of participants included 123 healthy individuals and 223 IC individuals before receiving the third dose of the SARS-CoV-2 vaccine. The final number of participants who underwent the sVNT test 3–6 months after the third dose was 60 in the healthy group and 141 in the IC group (41 HM, 49 SM, 23 RD, and 28 KT), accounting for losses to follow up. Among these participants, cellular immune responses were measured using IGRA in all 60 healthy individuals and 139 of the 141 IC individuals (*n* = 199 in total). A total of 3 of the 139 IC individuals had indeterminable results; therefore, the data of 136 participants were analyzed (n = 196 in total, including healthy individuals).

The results of T-cell immune responses in healthy and IC individuals showed that 83 of 196 (42.3%) participants tested negative in the IGRA using the tube coated with wild-type/alpha SP, whereas 96 of 196 (49%) participants tested negative in the IGRA using beta/gamma SP. Individuals with a negative IGRA to wild-type/alpha SP tended to be older on average, and most had underlying diseases (diabetes, hypertension, cancer, and chronic kidney disease; Table 1). In the comparison between healthy and IC individuals, 85% of healthy individuals and 45.6% of IC patients were IGRA-positive. The percentage of IGRA-negative results was higher in patients who underwent SM (*p* = 0.01) or KT (*p* < 0.001; Table 1). Regarding the vaccine type, the rate of IGRA (wild-type/alpha SP) positivity was higher in individuals who received an mRNA/mRNA/mRNA vaccine than in those who received an adenovirus vector/adenovirus vector/mRNA vaccine (Ad/Ad/mRNA; *p* = 0.002; Table 1). In the univariate analysis of the risk factors for negative IGRA results, the OR in patients with underlying diseases, such as diabetes, hypertension, cancer, and chronic kidney disease, was 5.883 (95% CI 2.929–11.819; *p* < 0.001; Table 2). The OR of IGRA-negative results was 0.148 in healthy individuals and 6.8 (3.085–14.826) in IC individuals (*p* < 0.001). The OR was 2.38 in patients who underwent SM (*p* = 0.001) and 6.04 in patients with KT (*p* < 0.001). Furthermore, the OR increased 2.7-fold in the adenovirus vector/adenovirus vector/mRNA vaccine group but decreased 0.4-fold in the mRNA/mRNA/mRNA vaccine group (*p* = 0.002; Table 2). In the multivariate analysis, which included variables with a *p* < 0.1 in the univariate analysis, the OR for IGRA-negative results was 4.7 in patients with an underlying disease (*p* = 0.045), while it decreased to 0.4 in the mRNA/mRNA/mRNA vaccine group (*p* = 0.045; Table 2).

In the univariate analysis of IGRA-positive results for beta/gamma SP, underlying disease (hypertension, cancer, and chronic kidney disease), current status (healthy or IC), and vaccine type were identified as factors influencing the rate of IGRA positivity (Table 2). The OR of negative IGRA (beta/gamma SP) was 3.6 in patients with an underlying disease (*p* = 0.045). Furthermore, a 1% increase in the monocyte% led to a 0.87-fold decrease in the rate of negative IGRA results (*p* = 0.008; Table 2).

The sVNT, conducted using the GenBody rapid kit and wild-type SP, showed that only 12 of 191 (6.3%) individuals tested negative (Table 3). In the univariate analysis of risk factors for the lack of neutralizing antibodies, underlying diseases, such as chronic kidney disease, and current status (healthy or IC) were identified as influencing factors. The OR for sVNT-negative results increased to 11 in patients with KT (*p* < 0.001) and to 3.4 in the adenovirus vector/adenovirus vector/mRNA vaccine group (*p* = 0.043). In the multivariate analysis, using variables with *p* < 0.1 from the univariate analysis, the OR of sVNT-negative results increased to 14 in patients with KT (*p* = 0.01; Table 4).

A risk factor analysis using PRNT_50_ was performed on 46 random samples. A total of 6 (13%) individuals tested negative when the wild-type virus was used, and 19 (41.3%) individuals tested negative when the omicron virus was used.

Regarding PRNT with the wild-type virus, a negative PRNT result of <1:8 was found in 33.3% of patients with chronic kidney disease (*p* = 0.015) and 36.4% of patients with KT (*p* = 0.01; Table 5). In the univariate analysis, the OR for the risk of PRNT-negative results increased 8-fold in patients with chronic kidney disease and 9.4-fold in patients who underwent KT (*p* < 0.05). In the multivariate analysis, the OR for PRNT-negative results was 8.7-fold higher in patients with KT (*p* = 0.049; Table 6). Regarding PRNT with the omicron virus, the OR for PRNT-negative results was higher in patients with underlying diseases, such as hypertension, chronic kidney disease, and KT. Furthermore, the OR was higher in the adenovirus vector/adenovirus vector/mRNA vaccine group than in the mRNA/mRNA/mRNA vaccine group (*p* = 0.035; Table 6). In the multivariate analysis, which included variables with a *p* < 0.1 in the univariate analysis, the OR for PRNT-negative results for the omicron virus increased 8.2-fold in patients who underwent KT (*p* = 0.039).

## 4. Discussion

During the COVID-19 pandemic, the reported COVID-19-related mortality rate in IC individuals with conditions such as solid organ transplantation, those on immunosuppressant medication, and those with HIV or congenital immunodeficiency ranged between 20 and 29% [14,15]. Reduced immune responses to SARS-CoV-2 after vaccination were associated with higher mortality or higher proportions of intensive care admissions in IC individuals with SM, HM, solid organ transplantation, or immunosuppressant use [3,16].

In a study involving patients with organ transplantation, antibodies were not produced in 80% of patients after two doses of the SARS-CoV-2 vaccine, and even after the third booster shot, 53% of patients remained antibody-negative [17]. Among those KT patients, non-seroconversion after the mRNA-1273 SARS-CoV-2 vaccine (Moderna) based on anti-spike IgG was 43.1% after the second dose and 25.6% after the third dose [11].

Patients with kidney failure are known to be at increased risk for SARS-CoV-2 infection, highlighting the critical need for effective vaccinations [18]. The humoral immune response is adequate in patients undergoing hemodialysis or peritoneal dialysis, but non-responders have been reported in some cases [7,19]. In a study involving 281 patients enrolled from five dialysis centers in northern Poland, cellular immunity was higher in patients with a pre-vaccination history of SARS-CoV-2 infection. The positive cellular response to vaccination was a positive factor to reduce all-cause mortality [16]. Therefore, assessing patient-related risk factors is critical for a lack of cellular or humoral immune responses.

In the present study, IC patients more frequently exhibited a lack of cellular immune response. Specifically, patients with SM or KT showed a greater percentage of IGRA-negative results. This finding is consistent with a study among 209 patients after two doses of the mRNA vaccine where positive IGRA was documented in 89.3% on peritoneal dialysis, 77.6% on hemodialysis, 61.3% of KT patients more than 1 year post-transplant, and only 36% in those transplanted within past 12 months [20]. Patients with malignancy of the lung, breast, colon, bladder, head–neck, prostate, rectum, and esophagus were found to have lower percentage of CD4^+^ and CD8^+^ T cells compared to those who had received four doses of the mRNA vaccine or those patients on hemodialysis [9].

GenBody rapid kit-based sVNT using wild-type SP showed that only 12 of 191 (6.3%) individuals tested negative, indicating that most individuals developed neutralizing antibodies against the wild-type virus. This is similar to a study among healthy individuals based on sVNT in Thailand where receiving two or three heterologous boosters with DNA- and/or mRNA vaccines was highly effective against Wuhan Hu-1 strain but had significant variations against the omicron variant [21]. However, those receiving three booster doses had higher levels of the neutralizing antibody than those who had received only two booster doses [21].

In our study, the PRNT was not performed for all patients, as it is a labor-intensive test. Only 6 of 46 (13%) individuals tested negative for the wild-type virus, whereas 19 (41.3%) individuals tested negative for the omicron virus on PRNT. These results suggested a significantly lower neutralizing antibody titer against the omicron variant than against the wild-type virus. This is similar to a study among IC individuals with cancer or hemodialysis, where the overall PRNT_50_ titer against omicron was lower than that against the Wuhan strain (*p* < 0.0001) [9]. Cancer patients with lung, breast, colon, bladder, head–neck, prostate, rectum, and stomach malignancies who had only three doses of the vaccine had a lower PRNT_50_ titer against omicron compared to those on hemodialysis, but there was no difference among those cancer patients who had received four doses when compared to those on hemodialysis [9].

Three to six months after vaccination, 22.2–29.6% of the KT patients in this study exhibited a cellular immune response. The percentage of PRNT-positive results was 63.6% against the wild-type virus but only 27.3% against the omicron virus, indicating that KT is a critical risk factor for impaired cellular or humoral immunity.

The risk factors associated with an impaired cellular immune response to beta/gamma SP were monocyte% and underlying diseases (hypertension, cancer, and chronic kidney disease). During acute SARS-CoV-2 infection, the number and function of immune cells, including T cells, natural killer cells, monocytes, and dendritic cells, decrease significantly [22,23]. Specifically, patients with COVID-19 experience a substantial reduction in monocyte counts compared to controls [22]. Peripheral blood monocytes are thought to contribute to immune responses against viral pathogens and select monocyte subsets may be related to disease outcomes [23,24]. However, further studies are required to investigate the impact of monocytes on the cellular immune response in the context of immunization via COVID-19 vaccination as monocytes exposed to inactivated SARS-CoV-2 were found to secrete higher levels of IL-6, TNF-α, CXCL10, CXCL9, and CXCL11 upon restimulation [25].

According to a previous study, a more durable humoral response could be achieved when a third heterologous vaccination using a viral vector vaccine was administered after two doses of an mRNA vaccine compared to a homologous mRNA vaccine [26]. However, this study showed that the third heterologous vaccination using an mRNA vaccine after two doses of the viral vector vaccine resulted in a 40% PRNT-positive rate against the omicron virus, while three homologous doses of mRNA vaccines led to an 80% PRNT-positive rate. These findings suggest that homologous vaccination with mRNA may be more effective at eliciting humoral immunity. Based on the IGRA using the wild-type/alpha SP, analyzing risk factors for impaired cellular immune response revealed that the risk of impaired cellular immunity was higher with homologous doses of mRNA vaccines than with the heterologous third dose of an mRNA vaccine after two doses of viral vector vaccines. Thus, it can be hypothesized that homologous doses of mRNA vaccines might be more effective in patients with risk factors for an impaired cellular immune response, although further randomized controlled trials should be conducted as other studies suggest no differences in neutralizing antibody or T-cell response [21].

This study has several limitations. First, the neutralizing antibody titer using PRNT_50_ was not analyzed in all patients; instead, an indirect test using sVNT was conducted. However, both PRNT_50_ and sVNT identified KT as a critical risk factor for the lack of neutralizing antibody induction. Another limitation is that only the wild-type SARS-CoV-2 SP was used in the sVNT without testing the response to the omicron viral SP. In the IGRA, only the antigens of wild-type SARS-CoV-2 and the alpha, beta, and gamma variants were tested without evaluating the cellular immune response against the omicron virus.

## 5. Conclusions

In summary, this study showed that underlying diseases, such as hypertension, cancer, and chronic kidney disease, are risk factors for an impaired cellular immune response after the third dose of the SARS-CoV-2 vaccine. Furthermore, KT has been identified as a risk factor for impaired humoral immune response. Overall, these results suggest the requirement of precautions against impaired immune responses in patients with an underlying disease or a history of KT.

## Figures and Tables

**Table 1:** Comparison of the IGRA-positive and -negative groups against SARS-CoV-2 wild-type/alpha SP and beta/gamma SP in both healthy individuals and IC individuals.

Characteristics	IGRA Using Wild-Type/Alpha		*p* Value	IGRA Using Beta/Gamma		*p* Value
	Negative (n = 83)	Positive (n = 113)		Negative (n = 96)	Positive (n = 100)	
Male gender, N (%)	50 (60.2%)	66 (58.4%)	0.796	58 (60.4%)	58 (58.0%)	0.731
Age, median (IQR)	56.0 (49.0–67.0)	51.0 (39.0–60.5)	0.024	55.5 (48.0–65.0)	52.0 (38.25–62.0)	0.115
BMI, median (IQR)	22.9 (20.31–25.79)	23.14 (21.14–25.08)	0.574	23.15 (20.5–25.54)	22.86 (21.04–25.27)	0.701
Underlying diseases, N (%)	70 (56.5%)	54 (43.5%)	<0.001	73 (58.9%)	51 (41.1%)	<0.001
Cardiovascular disease, N (%)	9 (42.9%)	12 (57.1%)	0.960	10 (47.6%)	11 (52.4%)	0.895
Diabetes mellitus, N (%)	24 (58.5%)	17 (41.5%)	0.018	23 (56.1%)	18 (43.9%)	0.305
Hypertension, N (%)	40 (64.5%)	22 (35.5%)	<0.001	40 (64.5%)	22 (35.5%)	0.003
Chronic lung disease, N (%)	4 (80.0%)	1 (20.0%)	0.084	4 (80.0%)	1 (20.0%)	0.160
Cancer, N (%)	47 (53.4%)	41 (46.6%)	0.005	51 (58.0%)	37 (42.0%)	0.023
Chronic kidney disease, N (%)	21 (70.0%)	9 (30.0%)	0.001	20 (66.7%)	10 (33.3%)	0.035
Disease status			<0.001			<0.001
Healthy, N (%)	9 (15.0%)	51 (85.0)	<0.001	15 (25.0%)	45 (75.0%)	<0.001
IC, N (%)	74 (54.4%)	62 (45.6%)	<0.001	81 (59.6%)	55 (40.4%)	<0.001
Hematologic malignancy, N (%)	20 (48.8%)	51 (51.2%)	0.348	22 (53.7%)	19 (46.3%)	0.500
Solid malignancy, N (%)	27 (58.7%)	19 (41.3%)	0.010	29 (63.0%)	17 (37.0%)	0.029
Rheumatic disease, N (%)	6 (27.3%)	16 (72.7%)	0.129	11 (50.0%)	11 (50.0%)	0.919
Kidney transplantation, N (%)	21 (77.8%)	6 (22.2%)	<0.001	19 (70.4%)	8 (29.6%)	0.017
Vaccine type (1st/2nd/3rd)			0.002			0.043
Ad/Ad/mRNA, N (%)	35 (59.3%)	24 (40.7%)	0.002	35 (59.3%)	24 (40.7%)	0.057
mRNA/mRNA/mRNA, N (%)	28 (30.8%)	63 (69.2%)	0.002	36 (39.6%)	55 (60.4%)	0.014
Other, N (%)	20 (43.5%)	26 (56.5%)	0.859	25 (54.3%)	21 (45.7%)	0.405
CBC						
WBC (10^3^/mL)	5.5 (4.28–6.73)	5.7 (4.93–7.08)	0.407	5.5 (4.3–6.85)	5.7 (5.05–7.0)	0.250
Neutrophils (%)	55.55 (49.5–63.0)	54.05 (46.83–63.83)	0.426	55.5 (49.55–63.45)	54.0 (47.05–61.6)	0.563
Lymphocytes (%)	31.8 (23.85–39.75)	32.95 (24.65–39.78)	0.927	30.7 (23.7–39.8)	33.6 (25.7–39.55)	0.894
Monocyte (%)	8.4 (6.1–11.33)	8.45 (6.63–12.3)	0.103	8.3 (6.15–11.25)	8.5 (6.85–12.8)	0.060
Eosinophil (%)	2.0 (1.08–3.7)	2.25 (1.3–3.63)	0.551	2.0 (1.2–3.7)	2.2 (1.0–3.75)	0.871
Platelet (10^3^/µL)	217.0 (162.25–276.25)	230.0 (172.5–286.5)	0.644	218.0 (163.5–273.5)	231.0 (159.5–291.5)	0.492

Cf. IGRA (interferon-gamma release assay), IQR (interquartile range), BMI (body mass index), IC (immunocompromised host), CBC (complete blood cell), WBC (white blood cell).

**Table 2:** Risk factor analysis of the IGRA-negative results against SARS-CoV-2 wild-type/alpha SP and beta/gamma SP in both healthy individuals and IC individuals.

Characteristics	IGRA Using Wild-Type/Alpha				IGRA Using Beta/Gamma			
	Univariate Analysis		Multivariate Analysis		Univariate Analysis		Multivariate Analysis	
	Odds Ratio (95% CI)	*p* Value	Odds Ratio (95% CI)	*p* Value	Odds Ratio (95% CI)	*p* Value	Odds Ratio (95% CI)	*p* Value
Male gender, N (%)	1.079 (0.606–1.922)	0.796			1.105 (0.625–1.955)	0.731		
Age, median (IQR)	1.023 (1.003–1.043)	0.025	0.992 (0.967–1.018)	0.544	1.015 (0.996–1.035)	0.116		
BMI, median (IQR)	0.976 (0.899–1.060)	0.572			0.984 (0.907–1.067)	0.699		
Underlying disease total, N (%)	5.883 (2.929–11.819)	<0.001	4.712 (1.586–14.003)	0.005	3.049 (1.655–5.618)	<0.001	3.582 (1.031–12.441)	0.045
Cardiovascular disease, N (%)	1.024 (0.410–2.555)	0.960			0.941 (0.380–2.328)	0.895		
Diabetes mellitus, N (%)	2.297 (1.140–4.630)	0.020			1.435 (0.718–2.869)	0.307		
Hypertension, N (%)	3.848 (2.041–7.255)	<0.001			2.532 (1.358–4.723)	0.003		
Chronic lung disease, N (%)	5.671 (0.622–51.702)	0.124			4.304 (0.472–39.222)	0.195		
Cancer, N (%)	2.293 (1.285–4.092)	0.005			1.930 (1.091–3.414)	0.024		
Chronic kidney disease, N (%)	3.914 (1.687–9.083)	0.001			2.368 (0.045–5.368)	0.039		
Disease status								
Healthy, N (%)	0.148 (0.067–0.324)	<0.001			0.226 (0.115–0.446)	<0.001		
IC, N (%)	6.763 (3.085–14.826)	<0.001	2.193 (0.763–6.303)	0.145	4.418 (2.244–8.697)	<0.001	1.758 (0.530–5.830)	0.357
Hematologic malignancy, N (%)	1.391 (0.697–2.776)	0.350			1.267 (0.636–2.527)	0.501		
Solid malignancy, N (%)	2.385 (1.216–4.679)	0.011			2.113 (1.071–4.170)	0.031		
Rheumatic disease, N (%)	0.472 (0.176–1.265)	0.136			1.047 (0.431–2.542)	0.919		
Kidney transplantation, N (%)	6.040 (2.313–15.771)	<0.001			2.838 (1.177–6.840)	0.020		
Vaccine type (1st/2nd/3rd)								
Ad/Ad/mRNA, N (%)	2.704 (1.445–5.061)	0.002	0.790 (0.278–2.245)	0.659	1.817 (0.978–3.375)	0.059	0.766 (0.234–2.513)	0.660
mRNA/mRNA/mRNA, N (%)	0.404 (0.225–0.727)	0.002	0.380 (0.148–0.981)	0.045	0.491 (0.277–0.869)	0.015	0.500 (0.164–1.524)	0.223
Other, N (%)	1.062 (0.545–2.070)	0.859			1.325 (0.683–2.570)	0.406		
CBC								
WBC (10^3^/mL)	0.939 (0.811–1.088)	0.405			0.918 (0.792–1.063)	0.252		
Neutrophils (%)	1.011 (0.984–1.039)	0.424			1.008 (0.981–1.036)	0.560		
Lymphocytes (%)	0.999 (0.970–1.028)	0.926			1.002 (0.973–1.032)	0.893		
Monocyte (%)	0.935 (0.861–1.015)	0.109			0.927 (0.855–1.006)	0.068	0.867 (0.779–0.964)	0.008
Eosinophil (%)	0.942 (0.777–1.144)	0.548			1.016 (0.838–1.232)	0.870		
Platelet (10^3^/µL)	0.999 (0.995–1.003)	0.641			0.998 (0.997–1.003)	0.489		

Cf. IGRA (interferon-gamma release assay), IQR (interquartile range), BMI (body mass index), IC (immunocompromised host), CBC (complete blood cell), WBC (white blood cell).

**Table 3:** Comparison of the surrogate virus neutralizing test-positive and -negative groups.

Characteristics	Surrogate Virus Neutralizing Test		*p* Value
	Negative (n = 12)	Positive (n = 189)	
Male gender, N (%)	10 (83.3%)	107 (56.6%)	0.069
Age, median (IQR)	56.5 (37.25–70.25)	54.0 (44.5–64.0)	0.874
BMI, median (IQR)	21.57 (19.41–24.94)	23.0 (20.91–25.41)	0.207
Underlying disease total, N (%)	11 (8.6%)	113 (91.4%)	0.035
Cardiovascular disease, N (%)	2 (9.5%)	19 (90.5%)	0.468
Diabetes mellitus, N (%)	2 (4.8%)	40 (95.2%)	0.710
Hypertension, N (%)	7 (10.9%)	57 (89.1%)	0.042
Chronic lung disease, N (%)	0 (0%)	6 (100%)	0.531
Cancer, N (%)	5 (5.5%)	86 (94.5%)	0.796
Chronic kidney disease, N (%)	6 (18.8%)	26 (81.3%)	0.001
Disease status			<0.001
Healthy, N (%)	0 (0.0%)	60 (100%)	0.020
IC, N (%)	12 (8.5%)	129 (91.5%)	0.020
Hematologic malignancy, N (%)	2 (4.9%)	39 (95.1%)	0.741
Solid malignancy, N (%)	3 (6.1%)	46 (93.9%)	0.959
Rheumatoid disease, N (%)	0 (0%)	23 (100%)	0.199
Kidney transplantation, N (%)	7 (25%)	21 (75%)	<0.001
Vaccine type (1st/2nd/3rd)			0.102
Ad/Ad/mRNA, N (%)	7 (11.3%)	55 (88.7%)	0.033
mRNA/mRNA/mRNA, N (%)	3 (3.3%)	88 (96.7%)	0.146
Other, N (%)	2 (4.2%)	46 (95.8%)	0.546
CBC			
WBC (10^3^/mL)	5.45 (5.15–8.93)	5.65 (4.48–6.9)	0.376
Neutrophils (%)	53.95 (41.28–75.0)	54.9 (48.7–62.1)	0.801
Lymphocytes (%)	28.0 (19.63–43.65)	32.85 (24.58–39.93)	0.717
Monocyte (%)	10.35 (6.58–13.35)	8.4 (6.5–11.33)	0.318
Eosinophil (%)	1.2 (0.08–2.93)	2.2 (1.2–3.73)	0.083
Platelet (10^3^/µL)	168.0 (152.5–236.5)	225.5 (164.25–282.0)	0.283

Cf. IQR (interquartile range), BMI (body mass index), IC (immunocompromised host), CBC (complete blood cell), WBC (white blood cell).

**Table 4:** Risk factor analysis of the surrogate virus neutralizing test-negative group.

Characteristics	Univariate Analysis		Multivariate Analysis	
	Odds Ratio (95% CI)	*p* Value	Odds Ratio (95% CI)	*p* Value
Male gender, N (%)	3.832 (0.817–17.967)	0.088	1.928 (0.319–11.670)	0.475
Age, median (IQR)	1.003 (0.965–1.043)	0.874		
BMI, median (IQR)	0.886 (0.735–1.069)	0.206		
Underlying disease total, N (%)	6.769 (0.856–53.541)	0.070	1.263 (0.101–15.791)	0.856
Cardiovascular disease, N (%)	1.789 (0.365–8.779)	0.473		
Diabetes mellitus, N (%)	0.745 (0.157–3.537)	0.711		
Hypertension, N (%)	3.242 (0.987–10.646)	0.053		
Chronic lung disease, N (%)	0.0 (0.0-)	0.999		
Cancer, N (%)	0.855 (0.262–2.792)	0.796		
Chronic kidney disease, N (%)	6.269 (1.879–20.917)	0.003		
Disease status				
Healthy, N (%)	0.0 (0.0-)	0.997		
IC, N (%)	4.418 (2.244–8.697)	<0.001		
Hematologic malignancy, N (%)	0.769 (0.162–3.655)	0.741		
Solid malignancy, N (%)	1.036 (0.269–3.990)	0.959		
Rheumatoid disease, N (%)	0.0 (0.0-)	0.998		
Kidney transplantation, N (%)	11.2 (3.260–38.473)	<0.001	13.777 (1.852–102.499)	0.010
Vaccine type (1st/2nd/3rd)				
Ad/Ad/mRNA, N (%)	3.411 (1.038–11.210)	0.043	5.521 (0.780–39.087)	0.087
mRNA/mRNA/mRNA, N (%)	0.383 (0.10–1.457)	0.159		
Other, N (%)	0.622 (0.131–2.941)	0.549		
CBC				
WBC (10^3^/mL)	1.132 (0.861–1.489)	0.375		
Neutrophils (%)	1.008 (0.949–1.071)	0.799		
Lymphocytes (%)	0.988 (0.925–1.055)	0.715		
Monocyte (%)	1.068 (0.937–1.218)	0.322		
Eosinophil (%)	0.588 (0.314–1.099)	0.096	0.635 (0.310–1.302)	0.216
Platelet (10^3^/µL)	0.993 (0.981–1.006)	0.283		

Cf. IQR (interquartile range), BMI (body mass index), IC (immunocompromised host), CBC (complete blood cell), WBC (white blood cell).

**Table 5:** Comparison of the plaque reduction neutralization test (PRNT_50_)-positive and -negative groups against the SARS-CoV-2 wild-type (V clade (B lineage)) or omicron (GR clade (B.1.1.529 lineage)).

Characteristics	PRNT Using WT		*p* Value	PRNT Using Omicron		*p* Value
	Negative (n = 6)	Positive (n = 40)		Negative (n = 19)	Positive (n = 27)	
Male gender, N (%)	3 (50%)	19 (47.5%)	0.909	10 (52.6%)	12 (44.4%)	0.584
Age, median (IQR)	51.0 (45.0–74.0)	58.0 (51.5–70.5)	0.495	57.0 (52.0–72.0)	58.0 (47.0–71.0)	0.904
BMI, median (IQR)	21.12 (18.88–23.18)	23.25 (21.01–26.62)	0.217	22.9 (20.92–26.61)	22.66 (20.76–26.62)	0.899
Underlying diseases, N (%)	6 (17.1%)	29 (82.9%)	0.160	17 (48.6%)	18 (51.4%)	0.107
Cardiovascular disease, N (%)	0 (0%)	8 (100%)	0.228	3 (37.5%)	5 (62.5%)	0.810
Diabetes mellitus, N (%)	2 (15.4%)	11 (84.6%)	0.767	7 (53.8%)	6 (46.2%)	0.278
Hypertension, N (%)	5 (20.8%)	19 (79.2%)	0.101	14 (58.3%)	10 (41.7%)	0.014
Chronic lung disease, N (%)	0 (0%)	0 (0%)		0 (0%)	0 (0%)	
Cancer, N (%)	2 (10.0%)	18 (90.0%)	0.591	7 (35.0%)	13 (65.0%)	0.446
Chronic kidney disease, N (%)	4 (33.3%)	8 (66.7%)	0.015	8 (66.7%)	4 (33.3%)	0.038
Disease status			0.065			0.078
Healthy, N (%)	0 (0%)	9 (100%)	0.221	1 (12.5%)	7 (87.5%)	0.061
IC, N (%)	6 (16.2%)	31 (83.9%)	0.195	18 (48.6%)	19 (51.4%)	0.040
Hematologic malignancy, N (%)	2 (16.7%)	10 (83.3%)	0.692	4 (33.3%)	8 (66.7%)	0.467
Solid malignancy, N (%)	0 (0%)	8 (100%)	0.221	3 (37.5%)	5 (62.5%)	0.766
Rheumatic disease, N (%)	0 (0%)	6 (100%)	0.302	3 (50.0%)	3 (50.0%)	0.679
Kidney transplantation, N (%)	4 (36.4%)	7 (63.6%)	0.010	8 (72.7%)	3 (27.3%)	0.018
Vaccine type (1st, 2nd, 3rd)			0.810			0.035
Ad/Ad/mRNA, N (%)	2 (13.3%)	13 (86.7%)	0.968	9 (60.0%)	6 (40.0%)	0.073
mRNA/mRNA/mRNA, N (%)	2 (18.2%)	18 (90.0%)	0591	4 (20%)	16 (80.0%)	0.010
Other, N (%)	2 (10.0%)	9 (81.8%)	0.586	6 (54.5%)	5 (45.5%)	0.341
CBC						
WBC (10^3^/mL)	7.0 (5.85–9.95)	5.2 (4.4–6.3)	0.044	5.9 (4.83–7.98)	5.15 (4.48–6.2)	0.110
Neutrophils (%)	54.9 (38.2–66.2)	54.9 (51.2–69.6)	0.417	53.95 (48.03–68.75)	56.05 (52.2–69.38)	0.807
Lymphocytes (%)	36.0 (21.5–49.85)	30.6 (22.8–36.0)	0.321	34.0 (20.18–68.75)	30.35 (23.28–35.05)	0.493
Monocyte (%)	8.0 (6.95–12.1)	8.7 (6.3–12.1)	0.817	8.0 (5.38–10.95)	8.75 (7.0–13.15)	0.529
Eosinophil (%)	1.5 (1.1–2.95)	1.5 (0.9–4.1)	0.735	1.25 (0.83–3.33)	1.75 (1.2–4.18)	0.232
Platelet (10^3^/µL)	191.0 (184.0-)	207.0 (143.75–256.0)	0.743	198.0 (184.0–223.0)	208.0 (134.5–274.0)	0.790

Cf. IQR (interquartile range), BMI (body mass index), IC (immunocompromised host), CBC (complete blood cell), WBC (white blood cell).

**Table 6:** Risk factor analysis of the plaque reduction neutralization test (PRNT_50_)-negative group against the SARS-CoV-2 wild-type (V clade (B lineage)) or omicron (GR clade (B.1.1.529 lineage)).

Characteristics	PRNT Using WT				PRNT Using Omicron			
	Univariate Analysis		Multivariate Analysis		Univariate Analysis		Multivariate Analysis	
	Odds Ratio (95% CI)	*p* Value	Odds Ratio (95% CI)	*p* Value	Odds Ratio (95% CI)	*p* Value	Odds Ratio (95% CI)	*p* Value
Male gender, N (%)	1.105 (0.199–6.150)	0.909			1.003 (0.960–1.047)	0.901		
Age, median (IQR)	0.977 (0.917–1.042)	0.488			1.389 (0.428–4.510)	0.585		
BMI, median (IQR)	0.851 (0.657–1.102)	0.220			0.989 (0.842–1.163)	0.897		
Underlying disease total, N (%)	334,236,182.8 (0.0-)	0.999			4.250 (0.801–22.563)	0.089	2.573 (0.284–23.344)	0.401
Cardiovascular disease, N (%)	0.0 (0.0-)	0.999			0.825 (0.172–3.964)	0.810		
Diabetes mellitus, N (%)	1.318 (0.211–8.249)	0.768			2.042 (0.556–7.497)	0.282		
Hypertension, N (%)	5.526 (0.591–51.647)	0.134			4.760 (1.316–17.216)	0.017		
Chronic lung disease, N (%)								
Cancer, N (%)	0.611 (0.100–3.727)	0.593			0.628 (0.189–2.085)	0.447		
Chronic kidney disease, N (%)	8.00 (1.238–51.690)	0.029			4.182 (1.032–16.939)	0.045		
Disease status								
Healthy, N (%)	0.0 (0.0-)	0.999			0.132 (0.015–1.163)	0.068		
IC, N (%)	312,672,558.7 (0.0-)	0.999			7.579 (0.860–66.813)	0.068		
Hematologic malignancy, N (%)	1.500 (0.238–9.465)	0.666			0.633 (0.160–2.512)	0.516		
Solid malignancy, N (%)	0.0 (0.0-)	0.999			0.825 (0.172–3.964)	0.810		
Rheumatic disease, N (%)	0.0 (0.0-)	0.999			1.500 (0.268–8.383)	0.644		
Kidney transplantation, N (%)	9.429 (1.434–61.986)	0.020	8.679 (1.009–74.659)	0.049	5.818 (1.290–26.249)	0.022	8.217 (1.107–6.966)	0.039
Vaccine type (1st/2nd/3rd)								
Ad/Ad/mRNA, N (%)	1.038 (0.168–6.421)	0.968			3.150 (0.877–11.311)	0.079	1.297 (0.153–10.966)	0.811
mRNA/mRNA/mRNA, N (%)	0.611 (0.100–3.727)	0.593			0.183 (0.048–0.703)	0.013	0.129 (0.016–1.070)	0.058
Other, N (%)	1.722 (0.270–10.981)	0.565			2.031 (0.516–7.997)	0.311		
CBC								
WBC (10^3^/mL)	1.422 (0.981–2.062)	0.063	1.363 (0.887–2.094)	0.158	1.274 (0.936–1.732)	0.123		
Neutrophils (%)	0.966 (0.891–1.048)	0.410			0.993 (0.943–1.047)	0.801		
Lymphocytes (%)	1.043 (0.960–1.133)	0.317			1.020 (0.966–1.077)	0.483		
Monocyte (%)	0.972 (0.770–1.227)	0.811			0.950 (0.813–1.111)	0.520		
Eosinophil (%)	0.896 (0.483–1.661)	0.727			0.770 (0.503–1.179)	0.229		0.770 (0.503–1.179)
Platelet (10^3^/µL)	0.997 (0.977–1.016)	0.733			0.999 (0.990–1.008)	0.813		0.999 (0.990–1.008)

Cf. IQR (interquartile range), BMI (body mass index), IC (immunocompromised host), CBC (complete blood cell), WBC (white blood cell).

## Data Availability

The data presented in this study are available on request from the corresponding author due to (Ethical reasons).

## References

[1] To K.K.-W., Tsang O.T.-Y., Leung W.-S., Tam A.R., Wu T.-C., Lung D.C., Yip C.C.-Y., Cai J.-P., Chan J.M.-C., Chik T.S.-H. (2020). Temporal profiles of viral load in posterior oropharyngeal saliva samples and serum antibody responses during infection by SARS-CoV-2: An observational cohort study. Lancet Infect. Dis..

[2] Wölfel R., Corman V.M., Guggemos W., Seilmaier M., Zange S., Müller M.A., Niemeyer D., Jones T.C., Vollmar P., Rothe C. (2020). Virological assessment of hospitalized patients with COVID-2019. Nature.

[3] Zohar T., Loos C., Fischinger S., Atyeo C., Wang C., Slein M.D., Burke J., Yu J., Feldman J., Hauser B.M. (2020). Compromised Humoral Functional Evolution Tracks with SARS-CoV-2 Mortality. Cell.

[4] Dan J.M., Mateus J., Kato Y., Hastie K.M., Yu E.D., Faliti C.E., Grifoni A., Ramirez S.I., Haupt S., Frazier A. (2021). Immunological memory to SARS-CoV-2 assessed for up to 8 months after infection. Science.

[5] Cui Z., Luo W., Chen R., Li Y., Wang Z., Liu Y., Liu S., Feng L., Jia Z., Cheng R. (2023). Comparing T- and B-cell responses to COVID-19 vaccines across varied immune backgrounds. Signal. Transduct. Target. Ther..

[6] Wankhede D., Grover S., Hofman P. (2023). Determinants of humoral immune response to SARS-CoV-2 vaccines in solid cancer patients: A systematic review and meta-analysis. Vaccine.

[7] Galmiche S., Luong Nguyen L.B., Tartour E., de Lamballerie X., Wittkop L., Loubet P., Launay O. (2022). Immunological and clinical efficacy of COVID-19 vaccines in immunocompromised populations: A systematic review. Clin. Microbiol. Infect..

[8] Sanders J.-S.F., Bemelman F.J., Messchendorp A.L., Baan C.C., van Baarle D., van Binnendijk R., Diavatopoulos D.A., Frölke S.C., Geers D., GeurtsvanKessel C.H. (2022). The RECOVAC Immune-response Study: The Immunogenicity, Tolerability, and Safety of COVID-19 Vaccination in Patients With Chronic Kidney Disease, on Dialysis, or Living With a Kidney Transplant. Transplantation.

[9] Campagna R., Dominelli F., Zingaropoli M.A., Ciurluini F., Grilli G., Amoroso A., De Domenico A., Amatore D., Lia M.S., Cortesi E. (2024). COVID-19 vaccination in cancer patients: Immune responses one year after the third dose. Vaccine.

[10] Yang J., Lee K.W., Baek J.Y., Bae S., Lee Y.H., Kim H., Huh K., Cho S.Y., Kang C.-I., Chung D.R. (2023). Augmented humoral and cellular immunity against severe acute respiratory syndrome coronavirus 2 after breakthrough infection in kidney transplant recipients who received 3 doses of coronavirus disease 2019 vaccine. Am. J. Transplant..

[11] Frölke S.C., Bouwmans P., Messchendorp A.L., Geerlings S.E., Hemmelder M.H., Gansevoort R.T., Hilbrands L.B., Reinders M.E.J., Sanders J.-S.F., Bemelman F.J. (2022). Predictors of Nonseroconversion to SARS-CoV-2 Vaccination in Kidney Transplant Recipients. Transpl. Direct.

[12] Cohen B.J., Audet S., Andrews N., Beeler J. (2007). Plaque reduction neutralization test for measles antibodies: Description of a standardised laboratory method for use in immunogenicity studies of aerosol vaccination. Vaccine.

[13] Lee B., Ko J.-H., Lee K.H., Kim Y.C., Song Y.G., Park Y.S., Baek Y.J., Ahn J.Y., Choi J.Y., Song K.H. (2022). Estimation of SARS-CoV-2 Neutralizing Activity and Protective Immunity in Different Vaccine Types Using Three Surrogate Virus Neutralization Test Assays and Two Semiquantitative Binding Assays Targeting the Receptor-Binding Domain. Microbiol. Spectr..

[14] Kates O.S., Haydel B.M., Florman S.S., Rana M.M., Chaudhry Z.S., Ramesh M.S., Safa K., Kotton C.N., Blumberg E.A., Besharatian B.D. (2021). Coronavirus Disease 2019 in Solid Organ Transplant: A Multicenter Cohort Study. Clin. Infect. Dis..

[15] Turtle L., Thorpe M., Drake T.M., Swets M., Palmieri C., Russell C.D., Ho A., Aston S., Wootton D.G., Richter A. (2023). Outcome of COVID-19 in hospitalised immunocompromised patients: An analysis of the WHO ISARIC CCP-UK prospective cohort study. PLoS Med..

[16] Kwiatkowska E., Safranow K., Wojciechowska-Koszko I., Roszkowska P., Dziedziejko V., Myślak M., Różański J., Ciechanowski K., Stompór T., Przybyciński J. (2022). SARS-CoV-2 mRNA Vaccine-Induced Cellular and Humoral Immunity in Hemodialysis Patients. Biomedicines.

[17] Werbel W.A., Boyarsky B.J., Ou M.T., Massie A.B., Tobian A.A.R., Garonzik-Wang J.M., Segev D.L. (2021). Safety and Immunogenicity of a Third Dose of SARS-CoV-2 Vaccine in Solid Organ Transplant Recipients: A Case Series. Ann. Intern. Med..

[18] Rincon-Arevalo H., Choi M., Stefanski A.-L., Halleck F., Weber U., Szelinski F., Jahrsdörfe B., Schrezenmeie H., Ludwi C., Sattler A. (2021). Impaired humoral immunity to SARS-CoV-2 BNT162b2 vaccine in kidney transplant recipients and dialysis patients. Sci. Immunol..

[19] Longlune N., Nogier M.B., Miedougé M., Gabilan C., Cartou C., Seigneuric B., Del Bello A., Marion O., Faguer S., Izopet J. (2021). High immunogenicity of a messenger RNA-based vaccine against SARS-CoV-2 in chronic dialysis patients. Nephrol. Dial. Transpl..

[20] Crespo M., Barrilado-Jackson A., Padilla E., Eguía J., Echeverria-Esnal D., Cao H., Faura A., Folgueiras M., Solà-Porta E., Pascual S. (2022). Negative immune responses to two-dose mRNA COVID-19 vaccines in renal allograft recipients assessed with simple antibody and interferon gamma release assay cellular monitoring. Am. J. Transpl..

[21] Nithichanon A., Kamuthachad L., Salao K., Phoksawat W., Kamsom C., Wongratanacheewin S., Pipattanaboon C., Kanthawong S., Yordpratum U., Aromseree S. (2023). A two-arm analysis of the immune response to heterologous boosting of inactivated SARS-CoV-2 vaccines. Sci. Rep..

[22] Lagadinou M., Zareifopoulos N., Gkentzi D., Sampsonas F., Kostopoulou E., Marangos M., Solomou E. (2021). Alterations in lymphocyte subsets and monocytes in patients diagnosed with SARS-CoV-2 pneumonia: A mini review of the literature. Eur. Rev. Med. Pharmacol. Sci..

[23] Zhou R., To K.K.-W., Wong Y.-C., Liu L., Zhou B., Li X., Huang H., Mo Y., Luk T.-Y., Yeung P. (2020). Acute SARS-CoV-2 Infection Impairs Dendritic Cell and T Cell Responses. Immunity.

[24] Park J., Dean L.S., Jiyarom B., Gangcuangco L.M., Shah P., Awamura T., Ching L.L., Nerurkar V.R., Chow D.C., Igno F. (2023). Elevated circulating monocytes and monocyte activation in COVID-19 convalescent individuals. Front. Immunol..

[25] Cvetkovic J., Jacobi R.H.J., Miranda-Bedate A., Pham N., Kutmon M., Groot J., van de Garde M.D.B., Pinelli E. (2023). Human Monocytes Exposed to SARS-CoV-2 Display Features of Innate Immune Memory Producing High Levels of CXCL10 upon Restimulation. J. Innate Immun..

[26] Liu X., Munro A.P.S., Wright A., Feng S., Janani L., Aley P.K., Babbage G., Baker J., Baxter D., Bawa T. (2023). Persistence of immune responses after heterologous and homologous third COVID-19 vaccine dose schedules in the UK: Eight-month analyses of the COV-BOOST trial. J. Infect..

